# Junk food consumption in relation to menstrual abnormalities among adolescent girls: A comparative cross sectional study

**DOI:** 10.12669/pjms.38.8.6177

**Published:** 2022

**Authors:** Samreen Latif, Saima Naz, Shazia Ashraf, Saghir Ahmed Jafri

**Affiliations:** 1 Ms. Samreen Latif, MS in Food, Nutrition and Dietetics, Lecturer, Department of Clinical Nutrition, Nur International University, Lahore, Pakistan; 2 Dr. Saima Naz, PhD. in Food Technology, Assistant Professor, Department of Clinical Nutrition, Nur International University, Lahore, Pakistan; 3 Ms. Shazia Ashraf, MS Community Health and Nutrition, HOD/Assistant Professor, Department of Clinical Nutrition, Nur International University, Lahore, Pakistan; 4 Dr. Saghir Ahmed Jafri, PhD in Biochemistry, Dean of Basic Sciences, Nur International University, Lahore, Pakistan

**Keywords:** Junk food, Irregular menstrual cycle, Premenstrual disorders, Dysmenorrhea, Body mass index

## Abstract

**Objectives:**

The study was aimed at evaluating the association between junk food consumption and BMI of adolescent girls along with the menstrual abnormalities and to compare it with controls.

**Methods:**

A cross-sectional study was conducted among 200 girls between 13 – 19 years of age at Bahria International Hospital, Lahore based on self-administered questionnaire from July 2021 to September 2021. The total subjects were divided in two groups Viz; Group-A which comprised of 100 girls with menstrual abnormalities and Group-B included 100 girls without menstrual problem (control group). The data recorded on the questionnaire about the demographic profile, anthropometric measurements, menstrual cycle characteristics, and dietary habits was subjected to statistical analysis using SPSS version 20 and Chi-Square was used to test quantitative significance between the two groups.

**Results:**

The mean age of participants was 17.02±1.76 years. It was observed that 40% girls had irregular menstrual cycle, 56% girls were suffering from dysmenorrhea and almost all girls of Group-I were suffering from premenstrual dysfunctions. The current study found a non-significant difference between two groups with regard to body mass index (P≥0.05). Significant difference was observed between two groups (P ≤ 0.05) as junk food consumption was high in Group-A as compared to Group-B. However, no significant difference was found between Group-A and B in relation to the consumption of salty snacks and frozen meat items (P≥0.05).

**Conclusion:**

The results suggested that junk food consumption affects menstrual cycle negatively however more studies are needed to confirm the association of BMI, consumption of salty snacks and frozen meat items with menstrual abnormalities.

## INTRODUCTION

Adolescence is the very complex transitional phase of mental and physical development between childhood and adulthood and is characterized by immense hormonal changes. Among adolescent girls, the most striking change is the onset of menstruation.^1^ Deviancy from normal physiological process of menstruation which is regulated by female sex hormones can lead to menstrual abnormalities such as irregular menstrual cycle, dysmenorrhea and premenstrual symptoms.^2^-^4^

Menstrual abnormalities are common among adolescent girls that can lead to stressful conditions. Worldwide about 75% girls are suffering from menstrual disorders.^5^ According to a study conducted in Pakistan about 43% girls experience problem related to irregular menstrual cycle, 44.4% experienced dysmenorrhea during menstruation and 75% girls suffered from premenstrual symptoms.^6^

Generally, there are couple of causes leading to menstrual abnormalities, which fall under five main categories: ailment, the effect of medication, genetics, psychological stress and lifestyle factors.^7^ For menstrual cycle regularity, Body mass index plays a very important role. Consequently, for the regulation of normal menstrual cycle of adolescent girls, it is essential to be given healthy and well balance diet, for the maintenance of their normal BMI.^8^

Many studies have explored that the consumption of junk foods can lead to menstrual abnormalities. In one of these studies irregular menstrual cycle, premenstrual symptoms and dysmenorrhea can be caused by the consumption of junk food because these kind of foods are deficient in micronutrients.^9^ According to another study dietary habits is one of the probable influencers on women’s quality of life and health which can influence numerous symptom of menstrual abnormality.^10^ Girls who regularly eat junk food (foods with high amount of sugar, fat, calories, salt and have low nutrient content), has more prevalence of dysmenorrhea among those girls. Junk foods might disturb the metabolism of progesterone in the menstrual cycle as they are rich in saturated fatty acids.^11^ According to a study it was observed that junk food consumption is correlated with dysmenorrhea however no association was found between junk food consumption with premenstrual symptoms.^12^ Furthermore in a study it was proved that lifestyle patterns such as reducing physical activities and consuming junk food directly disturbing the menstrual cycle of girls.^4^ Hence, it is essential to endorse the health education programs which should include regular physical activity, promoting adequate dietary intake and mindfulness on menstrual hygiene in school level for cultivating the menstrual health. It is very important to improve menstrual health for avoiding the many future and present gynecological problems such as infertility, polycystic ovaries and obesity.

## METHODS

This cross sectional study was conducted in Gynae Department of Bahria International Hospital Lahore. Study duration was three months from July 2021 to September 2021. Total 200 Girls aged from 13 to 19 years and those willing to take part were included in this study. The mean age of participants was 17.02±1.76 years. The total subjects were divided in two groups Viz; Group-A which comprised of 100 girls with menstrual abnormalities and Group-B included 100 girls without menstrual problem (control group) Participants presented with serious illnesses, those on any kind of pills, as well as those who were not ready to provide information, were all excluded from this research study.

### Data Collection:

The data was collected through self-administered questionnaire that was organized into divisions such as baseline data e.g. age, anthropometric measurements (height, weight, BMI), menstrual pattern, and food frequency questionnaire to assess junk food consumption. Before collecting the data, all of the participants were communicated of the data’s privacy.

### Ethical Consideration:

Ethical approval (NIU-6-2021-IRB-101) was taken on 2^nd^ of July from NUR International University as well as permission letter was also taken from Bahria International hospital on 25^th^ July, 2021.

### Data Analysis:

SPSS version 20 was used for statistical analysis. Before detailed analysis, data was first amended. Frequencies and percentages were used for presenting the baseline data and prevalence rate. Chi-square test was applied to compare the junk food consumption and body mass index of adolescent girls having menstrual abnormalities and those who do not have menstrual abnormalities. P-value was set at < 0.05 as significant.

## RESULTS

The age of menarche of most of the participants was between 13-15 years of the whole sampling population. Table-I shows that the menstrual abnormalities in Group-I, such as irregular cycle, was present in 40% girls, premenstrual symptoms in 97% and dysmenorrhea was observed in 56% girls. Table-I

**Table-I:** Prevalence of menstrual abnormalities among adolescent girls

Variables		Group-I (With Menstrual Abnormalities) N (%)	Group-II (Without Menstrual Abnormalities) N (%)
Irregular Menstrual Cycle	Yes	40 (40%)	0 (0%)
	No	60 (60%)	100 (100%)
Dysmenorrhea	Yes	56 (56%)	100 (22%)
	No	44 (44%)	0 (0%)
Premenstrual Symptoms	Yes	97 (97%)	100 (100%)
	No	3 (3%)	0 (0%)
Family history of menstrual abnormalities	Yes	34 (34%)	20 (20%)
	No	66 (66%)	80 (80%)

Table-II Indicates that no significant association was found between body mass index and menstrual abnormalities (P≥0.05).

**Table-II:** Comparison of Body Mass Index between Group-I (with menstrual abnormalities) and Group-II (control group).

			Group		Total

			Group-I Abnormal Menstrual Cycle	Group-II Normal Menstrual Cycle	
BMI	Underweight (BMI < 18.9)	Count	26	25	51
		% within BMI	51.0%	49%	100.0%
	Normal (BMI 19 - 25)	Count	58	63	121
		% within BMI	47.9%	52.1%	100.0%
	Overweight (BMI 25 - 30)	Count	12	9	21
		% within BMI	57.1%	42.9%	100.0%
	Obese (BMI > 30)	Count	4	3	7
		% within BMI	57.1%	42.9%	100.0%
Total		Count	100	100	200
		% within BMI	50.0%	50.0%	100.0%

Chi-Square Tests					

Pearson Chi-Square			Value	df	Asymp. Sig. (2-sided)

			.798^a^	3	.850

The association of junk food consumption with menstrual abnormalities is shown in Table-III. In present study junk food items was categorized into nine different groups so deep analysis could be done. These groups are refined grains, salty snacks, sweet snacks, bakery items, frozen meat items, carbonated beverages, street food, fast food and processed drinks. Significant difference between two groups was observed with regard to junk food consumption. It was found that consumption of refined grains, sweet snacks, bakery items, carbonated beverages, fast food and processed drinks was strongly associated with menstrual abnormalities (P≤0.05). However, no association was found between the consumption of salty snacks and frozen meat items with menstrual abnormalities among adolescent girls (P≥0.05).Table-III.

**Table-III:** Comparison of junk food consumption between Group-I (with menstrual abnormalities) and Group-II (control group).

		Groups				

		Group-I (With Menstrual Abnormalities)		Group-II (Without Menstrual Abnormalities)		P value

		Freq.	% age	Freq.	% age	
Consumption of Refined grains	High Consumption	63	63.0%	43	43.0%	.011^*^
	Moderate Consumption	20	20.0%	37	37.0%	
	Low Consumption	17	17.0%	20	20.0%	
Consumption of Salty snacks	High Consumption	51	51.0%	41	41.0%	.150
	Moderate Consumption	32	32.0%	31	31.0%	
	Low Consumption	17	17.0%	28	28.0%	
Consumption of Sweet snacks	High Consumption	69	69.0%	28	28.0%	.000^*^
	Moderate Consumption	14	14.0%	33	33.0%	
	Low Consumption	17	17.0%	39	39.0%	
Consumption of Bakery items	High Consumption	46	46.0%	26	26.0%	.005^*^
	Moderate Consumption	13	13.0%	26	26.0%	
	Low Consumption	41	41.0%	48	48.0%	
Consumption of Frozen meat items	High Consumption	32	32.0%	20	20.0%	.152
	Moderate Consumption	17	17.0%	21	21.0%	
	Low Consumption	51	51.0%	59	59.0%	
Consumption of Carbonated beverages	High Consumption	53	53.0%	20	20.0%	.000^*^
	Moderate Consumption	17	17.0%	29	29.0%	
	Low Consumption	30	30.0%	51	51.0%	
Consumption of Street foods	High Consumption	59	59.0%	8	8.0%	.000^*^
	Moderate Consumption	15	15.0%	28	28.0%	
	Low Consumption	26	26.0%	64	64.0%	
Consumption of Fast food	High Consumption	50	50.0%	15	15.0%	.000^*^
	Moderate Consumption	14	14.0%	27	27.0%	
	Low Consumption	36	36.0%	58	58.0%	
Consumption of Processed drinks	High Consumption	32	32.0%	15	15.0%	.012^*^
	Moderate Consumption	27	27.0%	28	28.0%	
	Low Consumption	41	41.0%	57	57.0%	

## DISCUSSION

There are many studies regarding the prevalence of menstrual abnormalities among adolescent girls, yet not many studies have been done on their association with junk food consumption and BMI. The study junk food consumption and BMI in relation to menstrual abnormalities among adolescent girls was the first kind of study in Pakistan. As junk food consumption and body mass index are modifiable risk factors, so early interventions can prevent future gynecological issues. This study attempted to find out the association of junk food consumption and body mass index with menstrual abnormalities.

This study was done with 200 adolescent girls with mean age of 17.02±1.76 years. Most of the studies from various geographical areas of Pakistan showed the age of menarche stabilized at the age of 11 to 13 year.^13^,^14^ However recent study showed that most of the girls hit menarche when they were between 13-15 years old which is almost consistent with earlier reports of another study.^15^ The result suggested that the prevalence of premenstrual symptoms (98%) is the highest among all menstrual abnormalities, which is supported by the results of another study.^16^ Dysmenorrhea is commonly experienced by females during menstruation. Previous studies conducted in Pakistan found that the prevalence of dysmenorrhea was 91.5%-79.5%.^17^,^18^ However recent study report that 56% girls from Group-I were suffering from dysmenorrhea. Irregular menstrual cycle (40%) was commonly present among girls although its percentage is more as compared to other studies.^19^,^20^ Current study indicate non-significant association between menstrual abnormalities and family history of menstrual disorders. However, Sasikala has reported significant association between menstrual abnormalities and family history of menstrual disorders.^21^

To date many studies have explored the association of body mass index with menstrual abnormalities however there is contradiction in the findings of these studies. Recent study did not show significant association between body mass index and menstrual abnormalities which is supported by the outcomes of other studies. However, this finding is not in line with the findings of another study in which it was found that body mass index is correlated with menstrual abnormalities.^22^

The present study aimed at co-relating junk food consumption with menstrual abnormalities. For this purpose, junk food was categorized into nine major groups. A highly significant association was found between the consumption of refined grains, sweet snacks, bakery items, street food, carbonated beverages, frozen meat items and fast food consumption with menstrual abnormalities which is consistent with findings of other studies.^22^,^23^ However, Rupa and coworkers did not find significant association between junk consumption with menstrual abnormalities.^24^ An interesting finding of present study is that among all categories of junk food items no significant association was found between the consumption of salty snacks and frozen meat items with menstrual disorders. The findings corroborate with the opinion of Helwa and coworkers who did not find significant association of menstrual abnormalities with consumption of salty snacks. Contrary to this Tadakawa and coworkers found consumption of processed salty snacks is associated with menstrual abnormalities.^25^ However association of consumption of frozen meat items with menstrual abnormalities need further investigation.

### Limitations:

Adolescent girls experience hormonal changes due to their transition phase that might lead to menstrual irregularities for period of time. To address the association of junk food with irregular menstrual cycle, further study should be done among girls of various age groups. Waist to Hip ratio should be assessed along with BMI to determine the association of body fat with menstrual abnormalities. As sample size was small so further study should be done with larger sample size.This being a cross-sectional study, we can propose relationship, but not ascertain causality.

## CONCLUSION

. Excessive intake of junk food was significantly associated with menstrual abnormalities. However, consumption of salty snacks, frozen meat items and Body Mass Index had insignificant association with menstrual disorders. Menstrual cycle is a normal physiological process however deviation from its regularity can cause several health issues in future like polycystic ovarian syndrome, obesity, infertility and hyperlipidemia

### Recommendations

Comprehensive education programs on endorsing healthy eating behaviors should be emphasized to prevent menstrual abnormalities among young girls. More studies are needed to confirm the association between the consumption of salty snacks, frozen meat items and Body Mass Index with menstrual abnormalities.

### Author’s Contribution:

**SL:** Concept, design,, conducted research, data analysis and interpretation, revised article critically for intellectual content and final submission, accountable for all aspects of the work in ensuring that questions related to the accuracy or integrity of any part of the work are appropriately investigated and resolved.

**SN:** Concept, design, revised article for final submission.

**SA:** Concept, design, revised article critically for important intellectual content and final submission.

**SAJ:** Concept, design, data interpretation, final approval of the version to be published.
